# Single or Double-Lumen Aspiration Needle? Revisiting Choices for In Vitro Fertilization or Intracytoplasmic Sperm Injection—A Critical Review

**DOI:** 10.3390/life15091334

**Published:** 2025-08-22

**Authors:** Gopinath Muruti, Mohd Faizal Ahmad, Muhammad Azrai Abu, Nurul Ilani Abdul Latif, Abdul Kadir Abdul Karim

**Affiliations:** 1 Advanced Reproductive Centre, Hospital Canselor Tuanku Muhriz, National University of Malaysia, Kuala Lumpur 56000, Malaysia; gopinathmuruti1982@gmail.com (G.M.); drmohdfaizal@ukm.edu.my (M.F.A.); azraiabu1983@gmail.com (M.A.A.); nurul.ilani@hctm.ukm.edu.my (N.I.A.L.); 2 Department of Obstetrics & Gynaecology, Faculty of Medicine, National University of Malaysia, Kuala Lumpur 56000, Malaysia

**Keywords:** aspiration needle, oocyte retrieval, in vitro fertilization, intracytoplasmic sperm injection

## Abstract

Transvaginal ultrasound-guided follicle aspiration remains the gold standard for oocyte retrieval in assisted reproductive technology (ART). This procedure employs either a single-lumen aspiration needle (SLN) or double-lumen aspiration needle (DLN), both of which are effective modalities for oocyte retrieval. The primary objective of this review is to systematically compare the impact and clinical outcomes associated with the use of SLN versus DLN in women undergoing ART. A systematic literature search was conducted across two databases, PubMed and Google Scholar, encompassing publications from their inception until May 2025, and articles published in English. A total of five studies were included in the final analysis. The oocyte yield and the number of MII oocyte did not differ significantly between the groups. Procedural duration was markedly shorter in the SLN group compared to the DLN group. No significant differences were observed in procedure-related complications across groups. Two randomized controlled trials reported comparable fertilization rates and numbers of high-quality embryos between the two needle types. Additionally, clinical pregnancy rates, ongoing pregnancy rates, and live birth rates were similar between the SLN and DLN groups. In conclusion, the utilization ofan SLN for oocyte retrieval demonstrates comparable efficacy to that of a DLN.

## 1. Introduction

Since its introduction in 1985, transvaginal ultrasound-guided follicular aspiration has become the gold standard for oocyte retrieval in assisted reproductive technology (ART), offering superior safety and efficacy over earlier laparoscopic techniques [1,2]. Over the decades, continued innovation, particularly in aspiration needle design, has played a pivotal role in improving oocyte recovery rates [1].

**Figure 1:**
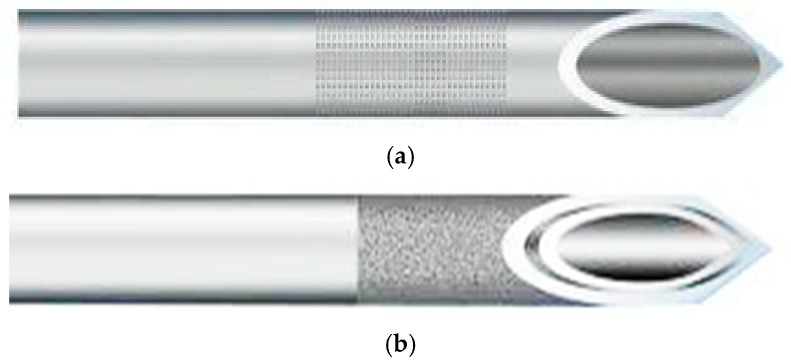
Schematic illustration of the anatomical differences in needle tip design between SLN and DLN. (**a**) Tip of single-lumen structure; (**b**) tip of double-lumen structure.

Currently, two principal needle types are used in ART procedures: the single-lumen aspiration needle (SLN) and the double-lumen aspiration needle (DLN) [3,4]. These devices differ not only in structure but also in procedural implications and clinical outcomes. Table 1 outlines their dimensional specifications, while Figure 1 illustrates the anatomical differences at the needle tip. The selection between SLN and DLN is typically driven by the clinical scenario and specific procedural goals. The SLN, equipped with a single channel for aspiration, enables a streamlined retrieval process, typically resulting in shorter procedure times [5]. In contrast, the DLN offers a more complex configuration, which may include either a single channel that alternates between aspiration and flushing (pseudo-double-lumen) or two distinct lumens—one for aspiration and another for follicular flushing [2,6]. Flushing theoretically increases oocyte yield by re-perfusing the follicle, potentially reducing oocyte retention and enhancing embryo production [7]. However, this presumed advantage remains contested. A study by Marcelo et al. (2021) found no statistically significant difference in the follicle-to-oocyte ratio between SLN and DLN among patients with poor ovarian response [2]. Moreover, several reports indicate that DLNs are associated with longer procedural durations, increased bleeding risk, greater invasiveness, and higher patient discomfort due to the added flushing step [1,2,6,7,8].

Understanding the fluid dynamics that underpin oocyte retrieval is crucial in interpreting these outcomes. Poiseuille’s law, which governs laminar flow through a cylindrical tube, provides insights into how lumen diameter, needle length, fluid viscosity, and applied vacuum pressure interact to affect the efficiency of follicular fluid aspiration [9,10]. These parameters are critical in optimizing needle design and enhancing oocyte retrieval systems in ART [11]. Despite the extensive clinical use of both needle types, a consensus on their comparative effectiveness remains elusive. Furthermore, with an increasing emphasis on individualized approaches in reproductive medicine, the question arises: does one needle type offer a tangible advantage over the other across different patient populations? This review aims to systematically evaluate and synthesize current evidence on the clinical efficacy, procedural efficiency, and safety profile of SLN versus DLN in oocyte retrieval. By integrating principles of fluid mechanics, clinical trial data, and practical considerations, we seek to inform best practices and identify areas for future innovation in ART instrumentation.

## 2. Materials and Methods

**Figure 2:**
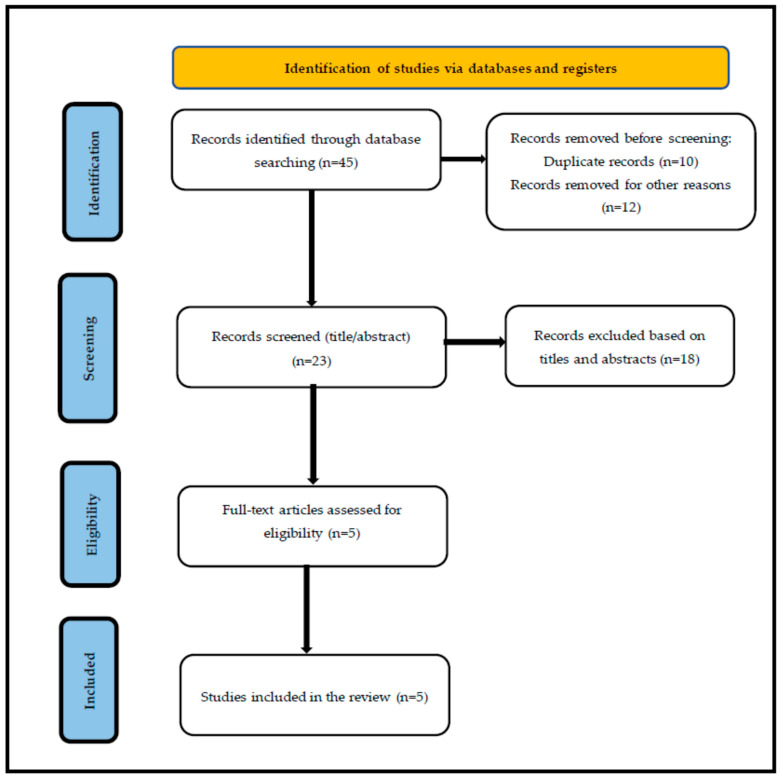
The process for the literature search.

This is a review of published data on SLN and DLN used for oocyte pick-up for in vitro fertilization or intracytoplasmic sperm injection (IVF/ICSI). We performed a systematic search by using two electronics databases (PubMed and Google Scholar). A literature search was performed from inception through May 2025 to identify relevant publications in English that addressed the utilization of the SLN and DLN in oocyte retrieval procedures. The search terms used alone or in combination were SLN, DLN, oocyte pick-up or oocyte retrieval, in vitro fertilization, and intracytoplasmic sperm injection. The inclusion criteria encompassed all experimental and observational studies published in English that provided relevant data on the advantages and disadvantages of using an SLN versus a DLN during oocyte retrieval. Only original research involving human subjects was considered eligible. Exclusion criteria included studies involving animal subjects, review articles, and case reports. Two reviewers performed the search, assessment, and data extraction processes. The first screening was based on the title and the abstract, and the second was on the evaluation of the full-text article. The bibliography was also analyzed to include articles that could have been missed. The most relevant articles were considered in this review. The selection and quality assessment of the included studies were independently conducted by three reviewers. Five studies were appraised using the Joanna Briggs Institute (JBI) Critical Appraisal Tools for randomized controlled trials (RCTs) and cohort studies. Each item on the checklist was scored as follows: ‘Yes’ = 1, ‘No’ = 0, and ‘Unclear’ = 0.5. The total score for each study was calculated as a percentage of the maximum possible score. Based on the final percentage score, studies were classified into three quality categories: <50% = poor quality, 50–69% = moderate quality, and >70% = high quality. The process for the literature search is shown in Figure 2. This review included five studies comparing SLN versus DLN in IVF/ICSI (Table 2).

## 3. Results

A total of 45 potentially relevant articles were retrieved from the search regarding the SLN and DLN system for IVF/ICSI, with 39 from Google Scholar and 6 from PubMed. A total of 10 duplicates were then removed. Another 12 studies were excluded due to animal subjects, review articles, and case reports. A total of five studies were deemed relevant to our objective. Full-text articles were obtained for all five studies. In this review, we analyzed data from the included studies to compare the SLN and DLN in terms of the total number of retrieved oocytes, number of mature oocytes (MII) obtained, procedure duration, procedural complications, fertilization rates, number of good quality embryos, clinical pregnancy rate, ongoing pregnancy rate, and live birth rate.

### Quality Assessment of Study Findings

Of the five studies reviewed, four were rated as high quality: Camilla R. et al. (2023) [1]—88%; Tian-JH et al. (2025) [5]—82%; Kyra von H. et al. (2017) [6]—92%; and Bulent H. et al. (2011) [8]—85%. One study was rated as moderate quality: Marcelo M. et al. (2021) [2]—69%.

## 4. Discussion

Although many clinicians recognize that the oocyte retrieval technique is crucial for IVF/ICSI and has been well described, the debate over whether to perform follicular flushing during oocyte retrieval remains unresolved. Despite the lack of strong evidence supporting the routine use of DLN with follicular flushing, it remains widely practiced in many ART clinics [1]. Uncertainty persists about its potential benefits for oocyte yield and treatment cycle outcomes [12,13].

### 4.1. Numbers of Retrieved Oocytes

A total of three studies showed no significant difference in the total oocyte yield. A randomized controlled trial conducted by Camilla R et al. (2023) [1], involving 200 oocyte retrieval procedures, found no significant difference in the number of oocytes retrieved between the SLN and the DLN groups (10.7 ± 7.0 vs. 10.2 ± 6.5, *p* = 0.810). Similarly, another randomized study by Bulent H et al. (2011) [8] reported no statistically significant difference in oocyte yield between the two groups (13.09 ± 4.45 for SLN vs. 12.25 ± 4.44 for DLN, *p* = 0.1). Kyra et al. (2017) [6], in another 80 procedures, also found that follicular flushing did not significantly improve the number of cumulus–oocyte complexes (COCs), with mean (SD) values of 2.4 (2.0) for the flushing group versus 3.1 (2.3) for the non-flushing group (*p* = 0.27).

In contrast, an Asian study published by Tian-J et al. (2025) [5] demonstrated a significant difference in oocyte retrieval, with the SLN yielding more oocytes compared to the DLN (12.84 vs. 11.56, *p* < 0.05). Although, on average, the difference is about one oocyte, due to the larger number of samples with an average oocyte yield of more than 10, there was statistical difference. This, however, did not translate to a difference in the MII collected. A difference was also found in a South American study by Marcelo et al. (2021) [2] in a randomized controlled trial involving 208 ovarian puncture procedures in patients with poor ovarian response, revealing that the SLN retrieved a higher number of oocytes compared to the DLN (3.69 ± 2.20 vs. 3.00 ± 2.11, *p* = 0.02). Although the average number of oocytes per patient was low, the difference remained statistically significant. In contrast to the other study of poor responders by Kyra et al. (2017) [6] no significant difference was found between the SLN and DLN groups.

Overall, the studies suggest that there is no clinical difference in the number of oocytes collected; however, in the poor ovarian responder cohort, the data does suggest that using an SLN is beneficial.

Although many fertility centers worldwide have ceased using follicular flushing in patients with a normal ovarian response, it continues to be employed in patients with poor ovarian response in an attempt to increase the number of oocytes retrieved.

### 4.2. Number of Mature (MII) Oocytes

In women with a normal ovarian response, three studies (Camilla R et al., 2023; Tian-J et al., 2025; and Bulent H et al., 2011) [1,5,8] reported comparable results regarding the number of mature oocytes (MII) retrieved between the SLN and DLN groups. In the cohort of poor responders, Kyra et al. (2017) [6] found no significant difference in the number of MII oocytes retrieved (2.1 vs. 1.6, *p* = 0.14), while Marcelo et al. (2021) [2] found a significant difference; however, the average numbers for both groups were less than three. The data suggest there is no clinical difference in choosing either an SLN or DLN in obtaining MII oocytes.

### 4.3. Procedure Duration

Four of the studies measured the duration of procedures. Camilla R et al. (2023) [1], Tian-J et al. (2025) [5], Kyra et al. (2017) [6], and Bulent H et al. (2011) [8] have demonstrated that the duration of the oocyte retrieval procedure is significantly shorter with the SLN compared to the DLN (single-lumen vs. double-lumen: 8.26 ± 2.99 vs. 12.52 ± 5.38 min, *p* < 0.05; 8.7 ± 3.3 vs. 11.1 ± 4.4 min, *p* < 0.001; 1.9 vs. 3.9 min, *p* < 0.001; and 14.23 vs. 17.68 min, *p* < 0.05, respectively). These findings suggest that the SLN is associated with a shorter procedural time, thereby reducing the need for anesthetic agents and minimizing the risk of anesthesia-related complications. Furthermore, the use of an SLN is more cost-effective for Assisted Reproductive Centers (ARCs), as it leads to significant reductions in procedural time, staffing requirements, consumption of flushing media, analgesics, and anesthetics, as well as lower overall production costs compared to the DLN [1,2,5,6,8]. Given these advantages, it is the authors’ view that the SLN approach could be routinely adopted in high-volume ART clinics, enhancing both economic and time efficiency while optimizing patient care, as supported by several previous studies.

### 4.4. Procedural Complications

Four studies briefly addressed procedural complications associated with oocyte retrieval. Camilla R et al. (2023) [1] reported an overall hospitalization rate of 0.29% within the first 24 h post-procedure across both the SLN and DLN groups, with a mean hospital stay of 2.77 ± 2.5 days. Only 0.1% of patients required urgent surgical intervention. However, specific types of complications were not detailed in this study.

In their research, Bulent H et al. (2011) [8] found no statistically significant difference in the incidence of ovarian hyperstimulation syndrome (OHSS) between the two groups, and no other procedural complications were reported.

Kyra et al. (2017) [6] found no significant differences in depression, anxiety, stress levels (as measured by the DASS-21), or pain scores within two hours post-procedure between the groups.

Additionally, the structural characteristics of the retrieval needles influence tissue trauma. Tian-J et al. (2025) [5] reported that under equivalent inner diameter conditions, the dual-layer configuration of the DLN results in a larger outer diameter, which may increase soft tissue damage. Conversely, SLNs with a smaller outer diameter are associated with reduced post-puncture pain and bleeding.

Based on the available evidence, there are no obvious differences in hospitalization rates within 24 h, OHSS, depression, anxiety, stress levels, or pain score between the SLN and DLN.

### 4.5. Fertilization Rates and Number of Good Quality Embryos

Three studies investigated this outcome. In two randomized controlled trials, fertilization rates, the number of cleaved embryos, total embryo count, the incidence of grade one embryos on day 3, and blastocyst formation rates were found to be comparable between SLN and DLN usage [1,8]. However, a retrospective cohort study conducted in Asia reported a higher normal fertilization rate (7.79 ± 5.1 vs. 6.98 ± 5.6, *p* = 0.043) in the SLN compared to the DLN group [5]. However, the good quality embryos for day 3 and day 5 did not differ significantly. All the aforementioned studies utilized flushing media at a frequency of one to three times, with individual flush volumes ranging from 1 to 3 mL per session. Several factors may contribute to the discrepancy in fertilization rate mentioned in the study by Tian-JH et al., 2025 [5]. Firstly, the number of flushes required during oocyte retrieval in the DLN group may play a critical role, as we observed flushing 1–3 times, and each time, there was a volume of 1–3 mL of flushing media. During repeated follicular flushing, the oocyte comes into direct contact with the flushing fluid. The oocyte is highly sensitive to environmental factors such as temperature, pH, osmotic pressure, and other variables, where even minor fluctuations can result in irreversible alterations. Repeated follicular flushing not only extends the duration of transvaginal aspiration but also increases the length of time the oocyte is immersed in the flushing solution, potentially leading to morphological abnormalities which can impair normal fertilization and a good quality embryo [7]. Furthermore, DLNs typically require higher negative pressure during oocyte retrieval, which may increase the risk of mechanical damage to oocytes, thereby compromising their quality [7,14]. Repetitive flushing also carries the potential for infection, as it may facilitate the pathogen contamination of oocytes and embryos, resulting in the absence of viable embryos. The author suggests that the adverse effects on the cumulus–oocyte complex and embryo quality may be minimized by employing an aspiration-only technique via the SLN route without follicular flushing.

### 4.6. Clinical Pregnancy Rate, Ongoing Pregnancy Rate, and Live Birth Rate

Three of the selected studies assessed outcomes after embryo transfer. One study looked at clinical pregnancy [5], two studies assessed ongoing pregnancy [1,5] and one study evaluated the live birth rate [8]. The results showed that none of these three parameters differ significantly. This analysis suggests that clinical outcomes are comparable between the SLN and DLN for oocyte retrieval, likely due to their shared fundamental function—aspiration of oocytes from ovarian follicles. When procedural techniques are standardized and optimized, potential discrepancies in outcomes can be minimized. Another potential reason is that the good quality oocytes are easier to retrieve. Studies have shown that the number of oocytes does not impact the pregnancy rates as larger follicles are more important [15]. Moreover, the experience and technical proficiency of the clinician performing the oocyte retrieval are critical factors that significantly impact clinical outcomes. The consistent application of best practices may result in similar outcomes, regardless of the type of needle used. Additionally, patient-specific factors, such as age, ovarian reserve, and response to ovarian stimulation protocols, play a pivotal role in determining the overall success of the treatment [16]. These individual variables may mask any differences in outcomes between needle types, particularly in patient populations exhibiting similar characteristics.

### 4.7. Benefits and Drawbacks of SLN and DLN

A well-executed technique using an SLN can result in the successful retrieval of a sufficient number of oocytes [5]. The SLN facilitates the dislodgement of the COC from the follicular wall through several mechanisms. First, follicular wall deformation caused by follicular fluid aspiration may induce oocyte dislodgement [17]. Second, the COC may be dislodged as it enters the beveled portion of the needle, where changes in velocity and direction create turbulence, further promoting the movement of the COC [3,18].

The DLN was introduced for oocyte retrieval with follicular flushing to reduce the risk of oocyte retention within the follicles or aspiration system. Its use is suggested to enhance the retrieval of oocytes during follicular aspiration [4,19,20,21]. The total number of oocytes retrieved is a critical prognostic factor in IVF [1]. The number of embryos obtained during ART cycles is directly correlated with the number of retrieved oocytes [19]. Moreover, live birth rates following IVF tend to increase with a higher number of retrieved oocytes, particularly when the count exceeds 15 [1,22]. In patients with a diminished ovarian reserve (DOR) or premature ovarian aging (POA), even a modest increase in the number of retrieved oocytes may significantly improve pregnancy outcomes [14,23,24].

The dual-layer design of the DLN increases the outer diameter while maintaining the same inner diameter, potentially leading to greater soft tissue injury. In contrast, an SLN has a smaller outer diameter, which may help minimize post-puncture pain and bleeding [25]. Additionally, the DLN, with its dual-layer structure, results in a smaller inner diameter for both the aspiration and flushing systems [26]. According to fluid dynamics principles, a smaller inner diameter necessitates higher pressure to achieve the same flow rate, which may compromise oocyte quality [27]. Conversely, the SLN features a relatively larger inner diameter, reducing the risk of clogging during oocyte retrieval, thereby enhancing the efficiency of oocyte collection [5].

### 4.8. Confounding Factors and Limitations of the Studies

All studies included patients with similar demographic characteristics, particularly in terms of age, body mass index (BMI), and duration of infertility between the SLN and DLN groups. This comparability helps to eliminate potential confounding related to age, BMI, and duration of infertility, thereby enhancing the internal validity of the studies. As a result, the consistency in these baseline characteristics contributes to the overall credibility and reliability of the reported outcomes. One potential confounding variable that may have influenced the results is operator experience performing oocyte pick-up. This factor was only reported in one study (Camilla R et al. (2023) [1]), while the remaining four studies did not address the experience level of the operator performing the oocyte pick-up. Operator experience can significantly affect key outcomes such as the number of retrieved oocytes, number of MII oocytes, procedure duration, and complication rates. The failure to account for this variable in most studies may have introduced bias, and thus, the reported outcomes should be interpreted with caution. A noteworthy confounding variable not addressed by the authors in two of the studies (Marcelo M et al., 2021 [2] and Tian-JH et al., 2025 [5]) was the specific type of ovarian stimulation protocol used, which may have influenced the study outcomes. Failure to account for this variable could have introduced bias into the results. The variation in stimulation protocols might partially explain the associations observed between the type of oocyte pick-up needle and the reported outcomes. In contrast, the remaining three studies clearly specified the stimulation protocols employed and the distribution of patients in each group (SLN vs. DLN), which, in the authors’ view, helps to minimize this potential confounding effect.

One of the limitations identified was the heterogeneity of the study cohorts regarding responder status. Among the four RCTs, two studies—Marcelo M et al., 2021 [2] and Kyra et al., 2017 [6]—were classified as involving poor responders. However, upon closer examination, the criteria used to define poor responders were not explicitly specified in study [2], and they did not conform to Bologna’s criteria for poor responders in a study [6]. The third study [1] did not specify the responder subgroup, while the fourth study [8] involved participants categorized as normal responders. This variability in cohort composition was considered a significant source of heterogeneity, leading to the conclusion that conducting a meta-analysis with these mixed cohorts would be inappropriate.

The findings of this systematic review should be interpreted with caution due to the limited number of studies that met our inclusion and exclusion criteria. A key limitation is the small number of published RCTs available on this topic. Nevertheless, we believe that the included studies—comprising four RCTs and one retrospective cohort study conducted across multiple centers—provide a meaningful representation of the current evidence base. To further validate the observed positive effects of SLNs and DLNs, future systematic reviews and meta-analyses incorporating a larger number of high-quality studies are warranted.

## 5. Conclusions

In conclusion, the utilization of an SLN for oocyte retrieval demonstrates comparable efficacy to that of a DLN. The clinical outcomes evaluated, including the total number of retrieved oocytes, mature oocytes (MII), fertilization rates, number of good quality embryos on day 3 and day 5, clinical pregnancy rates, ongoing pregnancy rates, and live birth rates, were statistically similar between both groups. Moreover, the SLN presents advantages in terms of cost-effectiveness and a reduction in procedural time.

## Figures and Tables

**Table 1:** Comparison of dimensional parameters between the SLN and DLN.

Needle Types	Single-Lumen Needle	Double-Lumen Needle
Principal/Function	Has one channel for follicular fluid aspiration	Has two channels: one for aspiration and one for flushing
Gauge Range/Options	16G to 21G	16G and 17G, commonly used
Outer Diameter	16G: corresponds to 1.6 mm 17G: corresponds to 1.4 mm 18G: corresponds to 1.2 mm 19G: corresponds to 1.1 mm 20G: corresponds to 0.9 mm 21G: corresponds to 0.8 mm	16G: corresponds to 1.65 mm 17G: corresponds to 1.5 mm
Inner Diameter	16G: corresponds to 1.4 mm 17G: corresponds to 1.2 mm 18G: corresponds to 1.0 mm 19G: corresponds to 0.8 mm 20G: corresponds to 0.6 mm 21G: corresponds to 0.6 mm	16G: corresponds to 1.0 mm 17G: corresponds to 0.9 mm
Needle Length	Commonly around 300–350 mm	Commonly around 300–350 mm
Aspiration/Flushing Tubing Length	Typically 900–1000 mm	Typically 900–1000 mm

**Table 2:** Summary of studies comparing SLN versus DLN in IVF/ICSI.

Author and Year	Title of Article/Study Design	Country	Sample Characteristics	Type of Needle/ Flushing Method	Follicle to Oocyte Index	Conclusions
**Bulent H et al., 2011 [8]**	In vitro fertilization–intracytoplasmic sperm injection outcomes in single versus double-lumen oocyte retrieval needles in normally responding patients: a randomized trial; prospective randomized	Turkey	Age—25 to 35 years SLN, *n* = 125 DLN, *n* = 149 Normal responder	SLN—17G Cook DLN—17G Cook DLN-flushing 1 time with 2 mL flushing media	Not mentioned	DLN did not demonstrate beneficial effect compared with SLN in terms of retrieved oocytes, clinical pregnancy rates, and live birth rates. DLN had a statistically longer duration of oocyte retrieval compared to SLN.
**Camilla R et al., 2023 [1]**	A Monocentric Randomized Controlled Clinical Trial to Compare Single and Double- Lumen Needles in Oocyte Retrieval Procedure in Assisted Reproductive Technologies; prospective randomized	Italy	Age—18 to 42 years SLN, *n* = 100 DLN, *n* = 100 Type of responder not mentioned	SLN—17G Cook DLN—17G Cook DLN-flushing 2 times, volume of flushing not mentioned	SLN—81% DLN—83%	No significant differences in terms of retrieval efficacy using SLN or DLN. DLN: significant increase in duration of the retrieval procedures.
**Kyra von H et al., 2017 [6]**	Randomized, open trial comparing a modified double-lumen needle follicular flushing system with a single- lumen aspiration needle in IVF patients with poor ovarian response; prospective randomized	Germany	Age—18 to 45 years SLN, *n* = 40 DLN, *n* = 40 Poor responder	SLN—17G Gynetics DLN-17G Steiner-Tan Needle flushing system DLN-flushing 3 times with flushing media, volume of flushing not mentioned	SLN—70% DLN—60%	No differences were observed in metaphase II oocytes, two pronuclear oocytes, number of patients having an embryo transfer, and Depression Anxiety and Stress Scale (DASS 21) scores. The procedure duration was significantly increased 2-fold in DLN.
**Marcelo M et al., 2021 [2]**	Evaluation of follicular flushing with double lumen needle in patients undergoing assisted reproductive technology treatments; prospective randomized	Brazil	Age—34 to 42 years SLN, *n* = 103 DLN, *n* = 105 Poor responder	SLN—19G Wallace DLN—17G Wallace DLN-flushing 2 times with half buffered medium, volume of flushing not mentioned	SLN—98% DLN—93%	No difference in the oocyte per follicle ratio, follicular flushing with DLN did not increase the number of oocytes recovered from poor responders. Recommended that direct aspiration with an SLN be used as the standard procedure for all patients.
**Tian-JH et al., 2025 [5]**	Introduction of the single- lumen oocyte retrieval needle in an assisted reproductive technology center: Propensity score matching analysis of grouping; retrospective cohort study (age, AMH, and body weight)	Taiwan	Age—31 to 41 years SLN, *n* = 228 DLN, *n* = 684 Type of responder not mentioned	SLN—18G KITAZATO DLN—17G Cook DLN-flushing 1–3 times, volume of 1–3 mL flushing media each time	Not mentioned	SLN reduced the procedure duration compared with DLN. SLN also increased the number of oocytes retrieved and the number of normal zygotes (2-PN) compared with DLN. Total number of viable embryos and the pregnancy rate per cycle demonstrates a favorable trend with SLN.

SLN—Single-lumen aspiration needle, DLN—double-lumen aspiration needle, and PN—Pronuclei.

## Abbreviations

The following abbreviations are used in this manuscript:

ART	Assisted reproductive technology
SLN	Single-lumen aspiration needle
DLN	Double-lumen aspiration needle
IVF	In vitro fertilization
ICSI	Intracytoplasmic sperm injection
PN	Pronuclei
COC	Cumulus-oocyte complex
MII	Mature oocyte
SD	Standard deviation
OHSS	Ovarian hyperstimulation syndrome
